# Knowledge and confidence of Australian emergency department clinicians in managing patients with mental health-related presentations: findings from a national qualitative study

**DOI:** 10.1186/1865-1380-6-2

**Published:** 2013-01-15

**Authors:** George A Jelinek, Tracey J Weiland, Claire Mackinlay, Marie Gerdtz, Nicole Hill

**Affiliations:** 1 Emergency Practice Innovation Centre, St. Vincent’s Hospital, Melbourne, Australia; 2 Faculty of Medicine, Dentistry and Health Sciences, The University of Melbourne, Melbourne, Australia; 3 School of Human Communication Science, La Trobe University, Melbourne, Australia; 4 School of Nursing and Social Work, Faculty of Medicine, Dentistry and Health Sciences, University of Melbourne and Nursing Research, Melbourne, Australia; 5 Honorary Senior Research Fellow, St. Vincent’s Hospital Melbourne, Melbourne, Australia; 6 ALERT Service, St. Vincent’s Hospital Melbourne, Melbourne, Australia

**Keywords:** Emergency department, Mental health, Learning needs analysis, Knowledge, Confidence

## Abstract

**Background:**

Mental health related presentations are common in Australian Emergency Departments (EDs). We sought to better understand ED staff knowledge and levels of confidence in treating people with mental health related problems using qualitative methods.

**Methods:**

This was a qualitative learning needs analysis of Australian emergency doctors and nurses regarding the assessment and management of mental health presentations. Participants were selected for semi-structured telephone interview using criterion-based sampling. Recruitment was via the Australasian College for Emergency Medicine and College of Emergency Nursing Australasia membership databases. Interviews were audio-recorded and transcribed verbatim. Thematic framework analysis was used to identify perceived knowledge gaps and levels of confidence among participants in assessing and managing patients attending EDs with mental health presentations.

**Results:**

Thirty-six staff comprising 20 doctors and 16 nurses consented to participate. Data saturation was achieved for four major areas where knowledge gaps were reported. These were: assessment (risk assessment and assessment of mental status), management (psychotherapeutic skills, ongoing management, medication management and behaviour management), training (curriculum and rotations), and application of mental health legislation. Participants’ confidence in assessing mental health patients was affected by environmental, staff, and patient related factors. Clinicians were keen to learn more about evidence based practice to provide better care for this patient group. Areas where clinicians felt the least confident were in the effective assessment and management of high risk behaviours, providing continuity of care, managing people with dual diagnosis, prescribing and effectively managing medications, assessing and managing child and adolescent mental health, and balancing the caseload in ED.

**Conclusion:**

Participants were most concerned about knowledge gaps in risk assessment, particularly for self-harming patients, violent and aggressive patients and their management, and distinguishing psychiatric from physical illness. Staff confidence was enhanced by better availability of skilled psychiatric support staff to assist in clinical decision-making for complex cases and via the provision of a safe ED environment. Strategies to enhance the care of patients with mental health presentations in Australian emergency departments should address these gaps in knowledge and confidence.

## Background

Mental health related problems are estimated to account for between 3 to 5% of Emergency Department visits in Australia [1-3]. In a recent mental health-related learning needs analysis of clinicians working in Australian public sector EDs, we reported a lack of knowledge and confidence in managing mental health related presentations. The gaps were very similar between emergency doctors and nurses, with nurses rating both knowledge and confidence lower than doctors [4]. Clinicians reported particular concern about managing patients presenting to EDs with personality disorder, psychosis or behavioural disturbance. They perceived knowledge deficits in developing care plans, conducting mental status examinations, assessing risk for self-harm, pharmacological management, responding effectively to patient aggression and alcohol or drug intoxication.

The current research was undertaken as part of a national qualitative survey of emergency nurses’ and doctors’ perceived learning needs regarding the assessment and management of patients with mental health conditions in Australian EDs. Findings from this study have previously been reported in relation to the optimal management of mental health related presentations, barriers to operation of mental health legislation, management of mental health conditions in rural and remote settings and the triage of mental health related problems [5-8]. In this particular study, we explore the perceived knowledge gaps of ED staff and the areas in which they lacked confidence in assessing and managing people presenting with mental health conditions. We aimed to use qualitative methods to more deeply explore issues around these clinicians’ knowledge and confidence in this area of emergency medicine.

## Methods

### Design

This was a qualitative learning needs analysis. Semi-structured telephone interviews were conducted with ED doctors and nurses. The research was approved by the Human Research and Ethics Committees (Faculty of Health Sciences) at La Trobe University and the Human Research Ethics Committee at St Vincent’s Hospital Melbourne. Study oversight was by a research team with meetings, and email contact, to oversee development of the interview schedule and data analyses. Detailed accounts of methods employed have previously been published [5-8].

### Participants

Participants working in a clinical role in an Australian ED and members of either the Australasian College for Emergency Medicine (ACEM; emergency doctors) or the College of Emergency Nurses Australasia (CENA; emergency nurses) were invited to take part in a telephone interview. We attempted to achieve proportional distribution of participants by location (metropolitan, regional/rural) and Australian states and territories by using a criterion-based sampling frame [9].

### Interview schedule

The research team including two emergency physicians, a researcher/emergency nurse, a research psychologist and a research officer used a consensus panel approach [9] to develop the semi-structured telephone interview schedule based on review of available literature. This included 16 open-ended interview questions to elicit participant’s views on a variety of clinician issues around mental health presentations to EDs. This part of the study examined the participants’ knowledge and confidence in assessing and managing people with mental health-related presentations in response to four specific questions.

1. What knowledge deficits do you feel that you or other emergency department clinicians have in respect to the assessment and management of mental health related presentations?

2. Do you think that emergency department clinicians would be interested in learning more about these conditions?

3. What factors affect your confidence in assessing and managing mental health related presentations?

4. In which areas do you feel least confident in assessing and treating mental health related problems?

### Recruitment

Emergency department clinicians were contacted via email. The initial invitation to participate was sent by ACEM and CENA to financial members. For those who expressed an interest, a plain language statement and participant information and consent form were emailed explaining the study in more detail. Following consent the interview questions were emailed in order to allow time to consider responses. Telephone interview responses were audio-recorded and transcribed verbatim. Transcripts were emailed back to participants to validate the credibility of the responses. One of the research team, a female researcher with qualifications in social work in an ED, and experience in qualitative methodologies conducted all telephone interviews.

### Analysis

Thematic analysis of the transcribed data was performed by two research officers using Spencer and Ritchie’s Framework method [9], resulting in a systematic thematic analysis of participant responses regarding perceived knowledge gaps regarding mental health-related presentations and the factors influencing their confidence in assessing and managing these presentations.

## Results

### Participant characteristics

The expression of interest emails resulted in 71 responses from ED doctors and nurses (39 ACEM and 32 CENA members), from which 20 ED doctors and 16 ED nurses were chosen to be interviewed on the basis of achieving a balanced sample from metropolitan and rural/regional EDs, the Australian states and territories, and ED seniority. Two thirds of the participants (24/36) worked in metropolitan EDs, just under one third in rural/regional locations (0/36) and 2 participants did not provide demographic data. One quarter of ED doctors were working as ED Directors/Deputy ED Directors (5/20), 8 were staff specialists and 7 were registrars. Table 1 shows the participants’ characteristics.

**Table 1:** Study participants’ characteristics by discipline, state or territory, and region

**Jurisdiction/Region**	Nurses (n=16*)		**Doctors (n=20)**	
	**Count**	**%**	**Count**	**%**
Victoria	4	29	5	25
New South Wales	0	0	6	30
Western Australia	4	29	3	15
Queensland	3	21	3	15
South Australia	3	21	1	5
Northern Territory	0	0	1	5
Tasmania	0	0	1	5
Australian Capital Territory	0	0	0	0
Metropolitan	11	79	13	65
Regional/Rural	3	21	7	35

*2 nurses provided no demographic data.

### Perceived knowledge gaps

Knowledge deficits were identified in four major areas. These were: assessment (risk assessment and mental state assessment), management (use of psycho-therapeutic skills, ongoing management, medication management and behaviour management), training (curriculum and rotations) and legislation.

Table 2 shows the major themes and sub-themes with sample quotes from study participants regarding the assessment and management of mental health presentations.

**Table 2:** Perceived knowledge gaps in the assessment and management of mental health related presentations reported by study participants

**Themes**		**Quotes**	
**Theme**	**Sub-theme**	**Doctors**	**Nurses**
Assessment	Risk assessment	*Making the suicide risk safety assessment [is a knowledge gap] if this person has come in saying they’re trying to kill themselves and I want to send them home. (M39)*	*I think the biggest knowledge deficit is that decision making on ‘are they safe to be discharged or are they not? (N4)*
	Mental status /describing symptoms	*Ability to perform a thorough mental state assessment is wanting… (M13)*	*It’s just the lack of knowledge on the types of illnesses, like what’s the real … difference between delusions and hallucinations and stuff, we get a bit stuck on. (N24)*
Diagnosis		*…to formulate a diagnosis or differential diagnosis can be problematic… (M13)*	*I think we didn't understand all the diagnoses. I think we would have loved a bit more training on different illnesses with mental health… (N10)*
Management	Psycho-therapeutic skills	*…the kind of brief intervention, for people who are not acutely disturbed, but need some counselling… (M29)*	*…[some staff] ignore problems and things will escalate and then all of a sudden you’ve got a violent patient on your hands… (N6)*
	Ongoing management	*…more to do with the ongoing management and medications and chronic effects of the illness rather than the acute presentation… (M31)*	*I acknowledge my level of knowledge is not high in terms of routine treatment and ongoing care plans.(N16)*
	Medications/Sedation	*….more info on the long-term side effects of medication.(M9)*	*…there’s a lot of debate over which is the best sedation agent to use. (N2)*
	Behaviour	*I feel pretty comfortable assessing mental health patients, but if they’re like really aggressive and hostile and distressed I find that difficult (M35)*	*…the immediate response to how you manage someone, to de-escalate the situation… (N20)*
Training	Curriculum	*Making sure that junior staff are up to date and know their stuff is actually very difficult. (M11)*	*Training in drug and alcohol issues would be good. (N8)*
	Rotations	*I think that ED registrars would also benefit from more psychiatric training. I did quite a bit of psych in my ED training. I think it’s something that people working in the ED probably could do with more training, particularly in the registrar phase when they are learning new skills anyway. (M7)*	*…mental health seems to get forgotten about yet we still have an expectation about staff that they’re competent and that they work in this mental health area. (N22*)
Legislation		*I think that we are not really formerly taught about the legal requirements about forms and transport issues (M33)*	

Assessment was a key theme for six doctors (M) and 13 nurses (N).

With respect to sub-themes, five doctors and nine nurses identified risk assessment as a key issue where they perceived knowledge levels to be inadequate. Specific knowledge about the effective use of assessment tools was identified by two doctors and six nurses.

….personally I could probably make use of [assessment] tools more. (M17)

Knowledge of current [assessment] tools is perhaps not what it should be. (M13)

Differentiating psychiatric disorders was identified as a theme by five doctors and three nurses. Similarly formulating differential diagnoses was raised as an area of concern as was dual diagnosis.

In terms of perceived knowledge gaps about the management of mental health related presentations, the use of brief psychotherapeutic interventions was mentioned by five doctors and one nurse.

…putting in place some strategies or some boundaries to say “ok, I know that you are upset, I hear what you’re saying, this is what I can do about it, but what I need you to do is this and this. That behaviour is not acceptable, if you do that this is what is going to happen”. People just really shy away from that. (N6)

Knowledge gaps regarding medication management were identified as an issue for 11 doctors and three nurses. Sedation in particular was identified as an area in which the participants felt they required greater knowledge.

I’ve never been properly taught in terms of who I should sedate, how I should sedate them (IV, IM, orally) and when/what the implications are for sedation so I think I would like to have some more guidelines for sedation. (M35)

Within this sub-theme four doctors identified new medications as a particular knowledge deficit.

Certainly things like new medications…, there’s obviously been a change in medication management… That sort of cutting edge stuff is stuff you hope you can stay abreast of, but trouble is often you don’t know what deficits you may or may not have. (M17)

Despite participating in education and training, six doctors and six nurses suggested behaviour management as a key area for improvement.

I did a one-day course on management of aggression training. It was quite useful about talking to people, and trying to talk them down. It’s something that certainly ED registrars would be good to have as part of their training… (N32)

I think often that needs more education - teaching and perhaps role-playing and that sort of thing to learn about managing… And trying to defuse violence… (M9)

Two doctors identified psychiatric training in general as a key theme, one identifying the trainee curriculum and the other rotation of junior medical staff as issues, and seven doctors reported knowledge gaps in understanding mental health legislation [6].

### Confidence

Participants’ confidence in assessing mental health patients was influenced by environmental factors (lack of resources and safety and security features of the environment), staff levels of experience in managing mental health problems, and case complexity (psychiatric/physical differentiation, accurate history, aggression, risk of self harm, dual diagnosis, personality disorder). Clinicians were keen to learn more about evidence based practice to provide better care for these patients.

Table 3 outlines the major themes and sub-themes on factors effecting confidence with sample quotes from study participants to further demonstrate these themes.

**Table 3:** Factors perceived by participants to influence confidence in the assessment and management of mental health related presentations

**Themes**		**Quotes**	
**Theme**	**Sub-theme**	**Doctors**	**Nurses**
Environment	Lack of resources	*When I work in the private system I have less confidence because I just don’t have the resources… (M7)*	*…everyone is time poor and the aim is to use whatever length of time in the most effective way. I think if opportunities are made available people will embrace them for sure. (N17)*
	Safety and security	*Certainly what increases my confidence is knowing that the environment is safe and secure. (M13)*	*Also there are times when I haven’t felt safe in a room where there’s only one exit and not many people around, it’s very closed off, the patient is aggressive, anxious and that affects me… (N24)*
Staff		*Most of my experience has been in city placements where there is a large frequent flow of psychiatric attendances. This regular contact helps to build confidence. (M33)*	*I guess I’ve had a lot of exposure in managing them, particularly when they are spectacularly off their tree. (N18)*
Case complexity		*…the other day we had a developmentally delayed patient, they’d had a brain injury 25-years ago, so they were actually quite difficult …. Ringing and talking it through with the psychiatrist made it pretty clear that he probably did have a mental illness… (M39)*	*…the dual diagnosis still does throw me. ...especially if they come in an they’re under the influence of the drugs or intoxicated… (N2)*
Behaviour		*People where you’re seeing an acute behavioural disturbance, and at the moment it doesn’t seem to belong clearly to either drug or alcohol or to psychiatry. I think in ED we get very caught around those issues. (M1)*	*…if they’re aggressive, I’m not going to ask them a lot of questions only because I’m worried about if I ask them something they don’t like, are they going to lunge at me, are they going to get crankier, so I don’t have a lot of confidence with psych patients at all… (N16)*

A total of 12 doctors and five nurses commented on lack of availability of resources affecting their confidence in treating mental health patients.

A secure and safe environment was a sub-theme for four doctors and five nurses.

Exposure to mental health patients and the clinician’s experience managing them was an important issue identified by nine doctors and four nurses.

…it’s really something to get used to over time. It really did used to make me quite nervous when I first started working in medicine, and I was much less confident in my decisions. I think it’s timely experience as much as anything… (M29)

Case complexity was also a factor reported to influence levels of confidence. In particular, differentiating psychiatric from physical illness presented a challenge for six doctors and three nurses.

…my confidence in diagnosis of psychiatric disorders in ED is extremely low, but I do not view that as my main role in ED. (M19)

Patient aggression was a significant factor affecting the confidence of two doctors and five nurses.

Aggression and anger is probably something I don’t deal with very well. (N16)

### Areas of least confidence

Areas where clinicians were least confident in treating mental health patients were described as problem/risky behaviours (personality disorder, risk assessment, self-harm), providing continuity of care (disposition, knowledge of available services), assessing and managing dual diagnosis, medications management, treating adolescent/children mental health problems, and balancing caseload in ED. Two doctors and two nurses expressed no lack of confidence in treating mental health patients.

Risk assessment was a particular concern for four doctors and two nurses who commented on the inconsistencies in the outcomes of risk assessments and associated dispositions.

The idea of someone who says that they can’t guarantee their safety but then making the call that they’re probably OK to go home. That’s one thing I’m less confident about. The . key . there is the risk associated with that… (M15)

Disposition decisions were an area of lack of confidence for four medical staff.

*…if they come in and they’re feeling a bit vulnerable and I actually don’t really feel they’re going to kill themselves, I do think they can go home back to the community …, I must say I always discuss it with a psychiatrist*… *(M19)*

In terms of specific diagnosis personality disorders was cited by five doctors and one nurse as the area in which they had the least confidence.

… personality disorders who are having a bit of a crisis – that’s still a difficult area for me… (M29)

…how to manage them [patients with personality disorders]can be difficult …. That’s one of the trickiest ones… (M21)

As an organisation and as a department we have huge issues managing people who have a long-standing personality disorder for example, and are presenting for treatment for an acute medical condition. (N16)

Knowledge of available services was cited as a cause of lack of confidence by four doctors, with these comments:

And also the knowledge of how to discharge patients into good outpatient care because there are a range of outpatient initiatives in our region… (M13)

I don’t know as much about community resources as I would like to (M17)

### Dual diagnosis

Dual diagnosis was a concern for four doctors and one nurse, particularly the combination of drug and alcohol problems with mental health issues.

I suppose probably the least confident is where there is the combination of drug and alcohol and mental health issues… (M5)

…especially if they come in and they’re under the influence of the drugs or intoxicated, it does make it difficult to make the right assessment and safety issues… (N2)

### Areas for education

In response to a question of whether the participant thought ED clinicians would be interested in learning more about particular areas, participants reported that clinicians were keen to learn more about evidence based best practice for these patients, in addition to issues around legislation, counselling, sedation, and differentiating between physical and psychiatric illness. Many clinicians (four doctors and five nurses) commented that this would depend largely on the personality of the clinician, and several (four doctors, one nurse) suggested that some attitudinal change might be necessary.

## Discussion

There is little literature on the knowledge and confidence of ED clinical staff in the management of patients with mental health-related presentations, with the available literature mostly relating to nurses. Small studies from the UK [10] and Australia [11] have shown that staff insecurities and culture can mean that mental health presentations have a low status in EDs compared with more dramatic physical illness, with ED nurses feeling a lack of skills and expertise to effectively manage this patient group. Cultural and systemic change with the introduction of psychiatric liaison nurses was shown to improve this situation [11], with staff responding favourably to this intervention. Importantly, nurses in the UK study identified a perceived deficit in mental health knowledge [10]. Participants in the Australian study expressed through questionnaire responses a particular lack of confidence in mental health triage, intoxicated and paranoid patients, and those resisting treatment, but also in assessment skills, particularly for self-harming patients [11]. The study reported a generalised lack of confidence in interacting with mental health patients.

One Western Australian study, stimulated by ED nurses having identified workplace safety and aggression as a key issue, showed through focus group discussions that ED nurses managing mental health patients identified customer focus, workplace aggression and violence, psychiatric theory, mental health assessment and chemical dependence as key learning areas [12]. The recruitment posters for this study were titled ‘What do ED nurses need/want in an education program to work effectively with aggressive or mental health presentations to ED?’ and this may have skewed learning needs identified towards safety and aggression, however other key issues identified in the focus groups were assessment and drug and alcohol issues. Of note, participants expressed deep concern about inadequate management of mental health patients in EDs, and felt that lack of knowledge was a key issue.

Our study has revealed an expressed range of perceived knowledge gaps and lack of confidence of clinicians of all levels of clinical experience working in Australian EDs in managing people with mental health-related presentations. In keeping with the findings of a quantitative national study of the same target group of clinicians [4], we found that risk assessment (notably for self-harming patients), the use of medications (particularly for acute sedation of agitated patients), behavioural disturbances (especially aggression and violence), and difficulties distinguishing psychiatric from physical illness to be areas that were problematic, both in terms of knowledge deficit and confidence.

Additionally, our study found that the confidence of clinical staff was enhanced by better availability of resources, mostly having access to other clinicians with greater knowledge and skill in managing mental health patients, but also in relying on other members of the ED clinical team, and security staff, for support. Security was an important issue affecting confidence, and a safe ED environment was seen as integral to optimal management, particularly with violent patients. ED clinicians were largely aware of their deficiencies, and noted the important contribution of experience to their confidence.

In line with the quantitative study, ED clinicians perceived risk assessment of self-harming patients, and behavioural disturbance, particularly in patients with personality disorders, as key areas where they felt the least confidence. These were areas which both ED medical and nursing staff rated the highest in terms of desired further training. Lack of knowledge of available community services however, and difficulties in making appropriate disposition decisions, were factors that were noted to affect confidence in our research, but were not reported in the quantitative study, highlighting the value of this qualitative research to better inform clinical service provision and education.

### Limitations

The sample included clinicians from most regions and grades of medical staff, but there were no nurses from New South Wales or clinicians from the Australian Capital Territory recruited for interview. This may limit the generalisability of our findings.

## Conclusions

Australian ED clinicians managing patients with mental health-related emergencies are most concerned about their knowledge deficits in risk assessment, particularly for self-harming patients, violent and aggressive patients and their management, and distinguishing psychiatric from physical illness. They report that their confidence improves with better access to trained psychiatric support staff and in a safe ED environment. Strategies to enhance the care of patients with mental health presentations should address these areas of deficit and discomfort.

## Competing interests

The authors declare that they have no competing interests.

## Authors’ contributions

All authors contributed to the study design, data analysis and writing of the paper, and have given final approval for publication of the paper. NH conducted the interviews.

## Funding

Funded by a competitive grant from the Windemere Foundation.
